# Color Restoration of RGBN Multispectral Filter Array Sensor Images Based on Spectral Decomposition

**DOI:** 10.3390/s16050719

**Published:** 2016-05-18

**Authors:** Chulhee Park, Moon Gi Kang

**Affiliations:** Department of Electrical and Electronic Engineering, Yonsei University, 50 Yonsei-ro, Seodaemun-gu, Seoul 03722, Korea; ascaron5@gmail.com

**Keywords:** color restoration, infrared cut-off filter removal, multispectral imaging, spectral estimation, spectral decomposition

## Abstract

A multispectral filter array (MSFA) image sensor with red, green, blue and near-infrared (NIR) filters is useful for various imaging applications with the advantages that it obtains color information and NIR information simultaneously. Because the MSFA image sensor needs to acquire invisible band information, it is necessary to remove the IR cut-offfilter (IRCF). However, without the IRCF, the color of the image is desaturated by the interference of the additional NIR component of each RGB color channel. To overcome color degradation, a signal processing approach is required to restore natural color by removing the unwanted NIR contribution to the RGB color channels while the additional NIR information remains in the N channel. Thus, in this paper, we propose a color restoration method for an imaging system based on the MSFA image sensor with RGBN filters. To remove the unnecessary NIR component in each RGB color channel, spectral estimation and spectral decomposition are performed based on the spectral characteristics of the MSFA sensor. The proposed color restoration method estimates the spectral intensity in NIR band and recovers hue and color saturation by decomposing the visible band component and the NIR band component in each RGB color channel. The experimental results show that the proposed method effectively restores natural color and minimizes angular errors.

## 1. Introduction

The near-infrared (NIR) is one of the regions closest in wavelength to the radiation detectable by the human eye. Unlike human eyes, sensors based on silicon (*SiO${}_{2}$*) are sensitive to NIR up to 1100 nm, limited by the cut-off value of silicon. Due to the proximity of NIR to visible radiation, NIR images share many properties with visible images. However, surface reflection in the NIR bands is material dependent. For instance, most dyes and pigments used for material colorization are somewhat transparent to NIR. This means that the difference in the NIR intensities is not only due to the particular color of the material, but also to the absorption and reflectance of dyes. Therefore, the NIR intensity provides the useful information pertinent to material classes rather than the color of that object [1].

Recently, there have been several attempts to use NIR band information. In remote sensing applications [2,3], the multispectral images observed in a variety of spectrum bands have been used where both the visible and NIR bands are included. As each spectral band provided different kinds of information, the spectral bands were selectively used in the observation of the multispectral images. In surveillance cameras [4] and night vision cameras [5], the NIR band is used especially under low lighting conditions or invisible NIR lighting conditions. The NIR band is also used in biometric [6], face matching [7] and face recognition [8] applications, which have been studied based on the intrinsic reflectivity of the skin or eyes under NIR illumination. Since the reflection in NIR is material dependent, it is also used in material classification [1] and illuminant estimation [9]. NIR images can be used in image enhancement applications, such as image dehazing [10].

To develop an NIR image acquisition system, Kise *et al.* designed a three-band spectral imaging system composed of multiple cameras with a beam splitter [11]. This imaging system has been used to acquire multispectral images in user-selected spectral bands simultaneously by utilizing three interchangeable optical filters and various optical components. Similarly, Matsui *et al*. implemented a multispectral imaging system, where two infrared cut-off filter (IRCF)-removed cameras were used to capture the color and NIR images independently [12]. In this system, the IRCF-removed cameras were perpendicularly aligned, and the IRCF was used as a light splitter for the visible and NIR bands. By managing the shutter of two cameras with a single controller, each spectral band image pair was acquired, simultaneously. However, this imaging system requires a large space to attach two or more cameras and to perform the alignment process. Due to the lack of portability of these devices, multi camera-based imaging systems are not suitable for practical outdoor environments. C. Fredembach [13] suggests another approach in which an IRCF-removed single camera with multiple optical band pass filters can achieve smaller sizes than multi-camera systems. On the other hand, this imaging system requires too much time to change the optical filters. Because of this weakness, some artifacts, like motion blur and registration problems, can occur during the image acquisition process.

**Figure 1:**
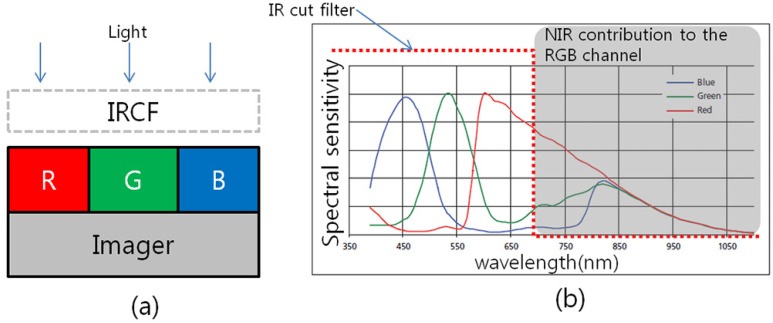
(**a**) Conventional camera system based on a color filter array (CFA) image sensor with the IR cut-off filter. (**b**) Spectral sensitivity of the camera system.

As an alternative approach, an IRCF-removed color filter array (CFA) image sensor, such as a Bayer image sensor without an IRCF, can be used [13]. By using a single digital camera without an IRCF, the spectral information of the visible bands and that of the NIR bands can be acquired at the same time. Figure 1 shows a conventional camera system approach with an IRCF and a spectral sensitivity of a complementary metal-oxide semiconductor (CMOS) imager integrated with traditional RGB Bayer filters. By removing the IRCF, the NIR contribution to the RGB channel can reach the CMOS imager. This additional NIR information can be used to allow for invisible monitoring in surveillance applications.

On the other hand, mixing color and NIR signals at the pixel level can result in extreme color desaturation if the illumination contains sufficient amounts of NIR. Although it may be possible to overcome the unwanted NIR contribution to the RGB color channel through the signal processing technique, it is hard to estimate the NIR spectral energy in each RGB color channel, because there is no way to detect the NIR band spectral characteristics.

**Figure 2:**
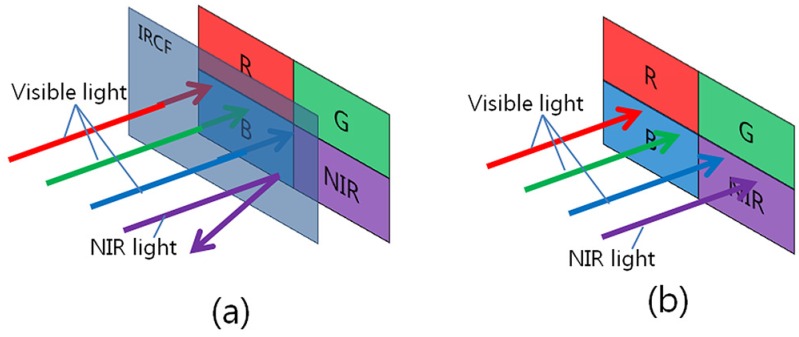
Infrared cut-off filter (IRCF): (**a**) typical imaging system using IRCF; (**b**) IRCF-removed imaging system.

As an improved system based on a single image sensor, an imaging system based on the multispectral filter array (MSFA), which simultaneously obtains visible and NIR band images, can be considered [14]. A pixel configuration of the RGB filters and another NIR pass filter, which transmits NIR light only, is shown in Figure 2. In the following descriptions, we refer to the four channels as RGBN channels, where RGB represents the red, green and blue channels and N represents the additional channel for the NIR band.

Because these sensors based on the RGBN filter array need to acquire invisible range information, removing IRCF is necessary. Without IRCF, RGB and NIR signals can be obtained simultaneously. Because of this advantage, imaging systems based on MSFA sensors can be applied to a wide variety of applications. Under certain circumstances, especially low lighting conditions, this system can obtain wide spectral information simultaneously. Furthermore, by applying fusion technology that uses NIR band information, gaining additional sensitivity to colors that do not deviate considerably from the human visual system is possible [15].

**Figure 3:**
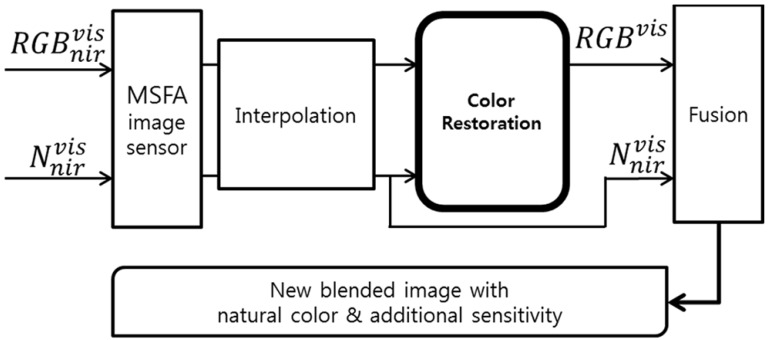
Example of a multispectral filter array (MSFA)-based imaging system.

However, without IRCF, the additional NIR component penetrates through the color filter to each R, G, B pixel. The unwanted NIR interference distorts the color information of each R, G, B color channel. Figure 3 is an example of an imaging system based on the MSFA image sensor. Many researchers studied the interpolation method, such as [15,16,17], to make a full resolution image in each RGBN channel. Since the input RGB signals contain NIR, natural RGB color information needs to be calculated by subtracting an NIR band component from the input RGB signals that have been deteriorated with NIR interference. During the process, the NIR channel information in the N pixel can be used to remove the unnecessary NIR contribution to the RGB channel. After restoring the color information of the RGB channel from the input signal received through the MSFA image sensors without an IRCF, a fusion method can be applied to generate the new blended images, which have not only natural color information, but also additional NIR spectral information. To take advantage of this benefit, it is necessary to restore natural color. As a result, the IRCF can be removed day and night with color restoration process.

In recent studies, researchers have proposed CFA for one-shot RGB and NIR capture in NIR imaging. However, the studies do not consider color restoration [16,17]. Although [18] addresses both crosstalk and demosaicing, it assumes crosstalk between the green and NIR channels only. Chen *et al.* proposed a color correction pipeline [15], which is able to apply only specific NIR illumination. The color correction method in [15] does not guarantee successful color correction results if the illumination spectrum is widely distributed in an NIR range. Furthermore, in [19], NIR restoration was proposed; however, the method does not consider crosstalk in a visible range in an NIR channel. The IR removal method proposed by Martinello *et al.* considers the crosstalk happening in the near IR range of 650 nm to 810 nm. This method assumes that the contribution from the wavelengths in the visible range (*λ* < 650 nm) to the IR channel can be ignored. On the contrary, the proposed color restoration method divides the visible and NIR bands to estimate the color correction matrix. In the visible band, the crosstalk in the N channel is estimated by using the linear regression of RGB channels by using the N channel decomposition matrix. By removing the estimated crosstalk in the N channel, the N channel information in the NIR band is obtained. The N channel information in the NIR band is used to estimate the NIR contribution to the RGB channels by using the RGB channel decomposition matrix. In this way, the proposed method copes with the different spectral responses of the visible and invisible bands, respectively. Furthermore, the proposed method considers the crosstalk happening in the near IR range from 650 nm to 1100 nm.

We proposed a brief idea to restore color information with an RGBN sensor [20]. However, since we focused only on color restoration under generally bright illumination environments, our previous work did not have good performance in low light conditions. In this paper, we proposes a color restoration method that removes the NIR component in each RGB color channel with an imaging system based on the IRCF-removed MSFA image sensor. To investigate the color restoration method for various illumination environments, we analyze the change of the chromaticity feature obtained by the additional NIR. In addition, the color restoration method for the low lighting condition based on the spectral energy distribution analysis is proposed. Since color degradation caused by IRCF removal is a huge limitation, the NIR contribution to each RGB color channel needs to be eliminated. To remove unwanted NIR components in each RGB channel, the color restoration model was subdivided into two parts of the spectral estimation and the spectral decomposition process.

The remainder of this paper is organized as follows: In Section 2, we discuss the problem that arises when a color image is acquired with the IRCF-removed MSFA image sensor. In Section 3, we analyze the color model of an IRCF-removed MSFA image sensor. In Section 4, we outline our proposed color restoration method with spectral estimation and spectral decomposition. In Section 5, we present our results and compare our solution to another state-of-the-art method. In Section 6, we provide a conclusion.

## 2. Color Degradation

To analyze the change of the chromaticity feature by the additional NIR, the RGB color space was converted to the HSI color space, as in [21]:

(1) $$\begin{array}{ccc} H & = & \cos^{-1}\left\{\frac{\frac{1}{2}\left[\left(R-G\right)+\left(R-B\right)\right]}{{\left[{\left(R-G\right)}^{2}+\left(R-B\right)\left(G-B\right)\right]}^{1/2}}\right\}\\ S & = & \frac{I-a}{I}\;\;\mathrm{where}\;a=min\left[\left(R,G,B\right)\right]\\ I & = & \frac{R+G+B}{3} \end{array}$$

where min[(·)] represents the minimum value among three values. *H*, *S* and *I* represent the hue, saturation and intensity, respectively.

**Figure 4:**
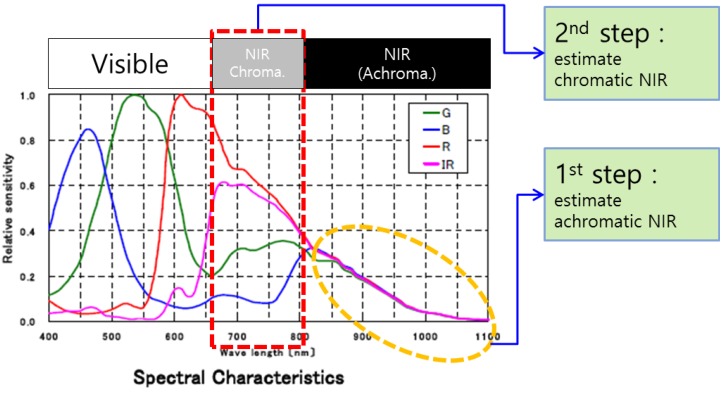
Spectral response of the MSFA image sensor.

**Figure 5:**
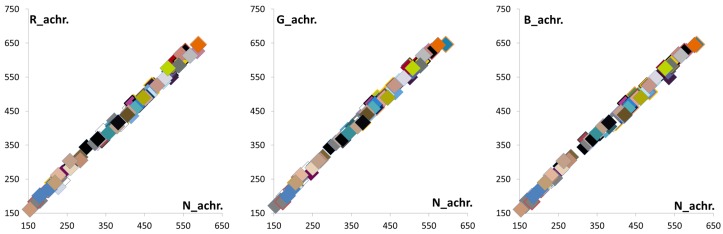
Correlation between the RGB channel and the N channel in the NIR band beyond 800 nm (**a**) $N_{nir(achr)}$ *vs.* $R_{nir(achr)}$ (**b**) $N_{nir(achr)}$ *vs.* $G_{nir(achr)}$ (**c**) $N_{nir(achr)}$ *vs.* $B_{nir(achr)}$.

In Figure 4, the NIR band is divided into two sub-bands: we define these sub-bands as a chromatic NIR band (700 nm∼800 nm) and an achromatic NIR band (800 nm∼1100 nm), respectively. Figure 5 shows that the responses of the achromatic NIR bands are identical. To obtain achromatic NIR band information, we used an NIR band pass filter that passes a specific wavelength (800 nm∼1100 nm). The distribution of 96 color patch values in the Gretag color checker SG shows a linear response in the achromatic NIR band with respect to the NIR channel. Based on this, we define these responses as a constant at each pixel, such as $R_{nir(achr)}=G_{nir(achr)}=B_{nir(achr)}=\delta$. The $R_{nir(achr)}$, $G_{nir(achr)}$ and $B_{nir(achr)}$ represent the achromatic colors of the image sensor beyond an 800-nm wavelength in each channel. As a result, the RGB intensities at a pixel position are represented as:

(2) $$\begin{array}{ccc} R(i,j) & = & R_{chr}\left(i,j\right)+\delta \left(i,j\right)\\ G(i,j) & = & G_{chr}\left(i,j\right)+\delta \left(i,j\right)\\ B(i,j) & = & B_{chr}\left(i,j\right)+\delta \left(i,j\right) \end{array}$$

where $R_{chr},G_{chr},B_{chr}$ represent the chromatic colors of the image sensor under an 800-nm wavelength.

With the RGB color values with offset *δ*, the intensity of the observed color is defined as follows:

(3) $$\begin{array}{ccc} I & = & \frac{\left[\left(R_{chr}+\delta \right)+\left(G_{chr}+\delta \right)+\left(G_{chr}+\delta \right)\right]}{3}\\ & = & I_{chr}+\delta \end{array}$$

where $I_{chr}=\left(R_{chr}+G_{chr}+B_{chr}\right)/3$ represents the intensity of the chromatic spectral band of the image sensor. The intensity of the IRCF-removed MSFA image sensor is changed by the amount of the offset value. The hue value in Equation (1) is redefined as:

(4) $$\begin{array}{cc} H= & \cos^{-1}\left\{\frac{\frac{1}{2}\left[\left(R-G\right)+\left(R-B\right)\right]}{{\left[{\left(R-G\right)}^{2}+\left(R-B\right)\left(G-B\right)\right]}^{1/2}}\right\}\\ = & \cos^{-1}\left\{\frac{\frac{1}{2}\left[\left(R_{chr}-G_{chr}\right)+\left(R_{chr}-B_{chr}\right)\right]}{{\left[{\left(R_{chr}-G_{chr}\right)}^{2}+\left(R_{chr}-B_{chr}\right)\left(G_{chr}-B_{chr}\right)\right]}^{1/2}}\right\} \end{array}$$

Because the achromatic offset value *δ* is removed during subtraction, an identical offset on the RGB channels could not change the hue value. Finally, the saturation value is described as:

(5) $$\begin{array}{ccc} S & = & \frac{I-a}{I}=\frac{I_{chr}-a_{chr}}{I}=\frac{I_{chr}}{I}\cdot S_{chr} \end{array}$$

where $S_{chr}=\left(I_{chr}-a_{chr}\right)/I_{chr}$ represents the saturation of the chromatic spectral band of the image sensor and $a_{chr}=min\left(R_{chr},G_{chr},B_{chr}\right)$. Since the range of $\frac{I_{chr}}{I}$ is $0\leq \frac{I_{chr}}{I}\leq 1$, the saturation of the image obtained by the IRCF-removed MSFA image sensor is degraded and becomes smaller than the image obtained by the chromatic spectral band of the image sensor.

**Figure 6:**
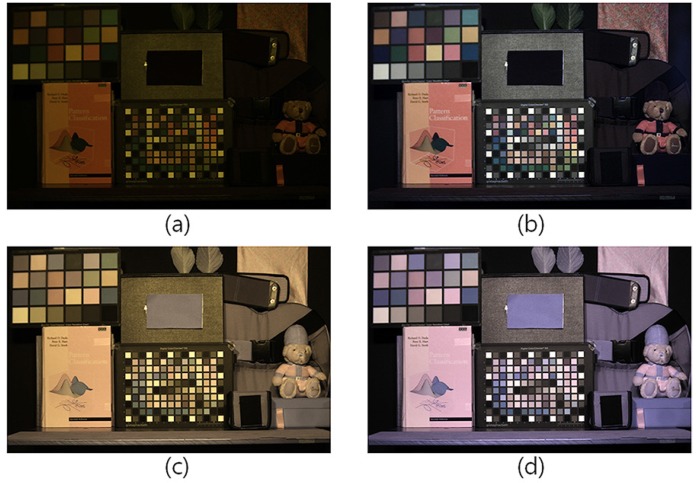
Color observation of the MSFA image sensor under incandescent light. (**a**) Image captured with IRCF; (**b**) (a) with white balance; (**c**) image captured with IRCF removal MSFA image sensor; (**d**) (c) with white balance.

Figure 6 describes how NIR affects the RGB color images. The illuminance was 200 lx, and the exposure time was 0.03 s. When objects are illuminated by an incandescent lamp, an image sensor with an IRCF obtains a yellowish hue due to the low color temperature of the illuminance. After performing a white balance technique from the grey color patch, a white-balanced color image was obtained as shown in Figure 6b. On the other hand, due to the additive NIR intensities included in the RGB channels, Figure 6c appears brighter than Figure 6a, and low color saturation was observed in Figure 6d.

To correct desaturated color from the input image acquired by the MSFA image sensor, several conventional methods can be considered, as described in [22,23]. A straightforward method is to train the matrix to reproduce a set of known reference colors. Given the observed color vector Y and the visible band color vector with canonical illuminance X, the color correction method is represented in a matrix form:

(6) $$X=\Phi^{T}Y$$

where Φ is a matrix whose component corresponds to the ratio between the canonical and the current illuminance value of each channel. The illuminant color estimation was performed under unknown lighting conditions where pre-knowledge based approaches, such as gamut mapping [24] or the color correlation framework [25], were used.

However, color degradation caused by IRCF removal is not considered a multiplicative process, but an additive process. Applying a conventional color correction approach to the RGBN images yielded poor results, because it did not sufficiently remove the NIR contributions to the RGB channels. The higher the energy in the NIR band relative to that in the visible band, the higher the color errors caused by NIR contributions to the RGB signals. As a result, the conventional color correction method restored visible band color in a limited way. Although each color was obtained under the same illuminant conditions with and without an IRCF, respectively, the mixture of the exclusive NIR band intensity to the visible band intensity resulted in severe color distortion.

**Figure 7:**
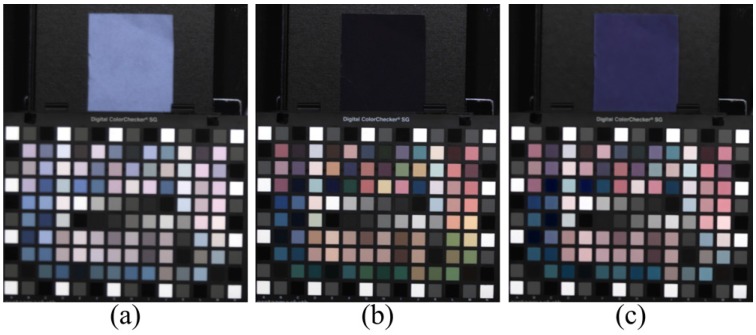
Example of the conventional color correction method for the MSFA image. (**a**) MSFA image without IRCF; (**b**) MSFA image with IRCF; (**c**) color correction result.

Figure 7 shows the result of the conventional color correction method for an MSFA image. In Figure 7c, the color correction matrix worked well for colors in the color chart with low reflectance in the NIR band. However, despite the fact that the colors of the black paper and velvet paper were the same in the visible band, the conventional color correction method could not restore the black color with high reflectance in the NIR band (such as fabric substance).

## 3. Color Model of an IRCF-Removed MSFA Image Sensor

A color image observed by a CMOS image sensor can be modeled as a spectral combination of three major components: illuminant spectra E(*λ*), sensor function $R^{\left(k\right)}\left(\lambda \right)$ and the surface spectra S(*λ*). The color image formation model in the visible band for channel *k* was defined as [26]:

(7) $$C_{vis}^{\left(k\right)}=\int_{w_{vis}}E\left(\lambda \right)R^{\left(k\right)}\left(\lambda \right)S\left(\lambda \right)d\lambda$$

where $w_{vis}$ represents the spectral range of the visible band between 400 nm and 700 nm. Since an IRCF-removed MSFA image sensor can acquire the additional NIR band spectral energy beyond a 700-nm wavelength, the range of these three major components in Equation (7) had to be expanded to the NIR band. The observed camera response for channel *k* when using the IRCF-removed MSFA image sensor is represented by the color image formation model $C_{MSFA}^{\left(k\right)}$ [19] from Equation (7):

(8) $$\begin{array}{ccc} C_{MSFA}^{\left(k\right)} & = & \int_{w_{vis}+w_{nir}}E\left(\lambda \right)R^{\left(k\right)}\left(\lambda \right)S\left(\lambda \right)d\lambda \\ & = & \int_{w_{vis}}E\left(\lambda \right)R^{\left(k\right)}\left(\lambda \right)S\left(\lambda \right)d\lambda +\int_{w_{nir}}E\left(\lambda \right)R^{\left(k\right)}\left(\lambda \right)S\left(\lambda \right)d\lambda \\ & = & C_{vis}^{\left(k\right)}+C_{nir}^{\left(k\right)} \end{array}$$

where $w_{nir}$ represents the NIR band beyond 700 nm. $C_{vis}^{\left(k\right)}$ and $C_{nir}^{\left(k\right)}$ represent the camera response for channel *k* by using the IRCF-removed MSFA image sensor in the visible band and the NIR band, respectively. For an image sensor with RGBN filters, the intensities at each pixel position are represented as,

(9) $$\begin{array}{cc} R(i,j) & =R_{vis}\left(i,j\right)+R_{nir}\left(i,j\right)\\ G(i,j) & =G_{vis}\left(i,j\right)+G_{nir}\left(i,j\right)\\ B(i,j) & =B_{vis}\left(i,j\right)+B_{nir}\left(i,j\right)\\ N(i,j) & =N_{vis}\left(i,j\right)+N_{nir}\left(i,j\right) \end{array}$$

In Equation (9), each pixel contained additional NIR band information. Since this additional information can be helpful to increase the sensitivity of the sensor, this feature can be useful under low light condition. However, mixing color and NIR intensities can result in color degradation if the illumination contains high amounts of NIR. To restore the RGB channels corrupted by NIR band spectral energy, the additional NIR band components ($R_{nir}$, $G_{nir}$, $B_{nir}$) in the RGB channels have to be removed:

(10) $$\begin{array}{cc} R_{vis} & =R-R_{nir}\\ G_{vis} & =G-G_{nir}\\ B_{vis} & =B-B_{nir}\\ N_{vis} & =N-N_{nir} \end{array}$$

**Figure 8:**
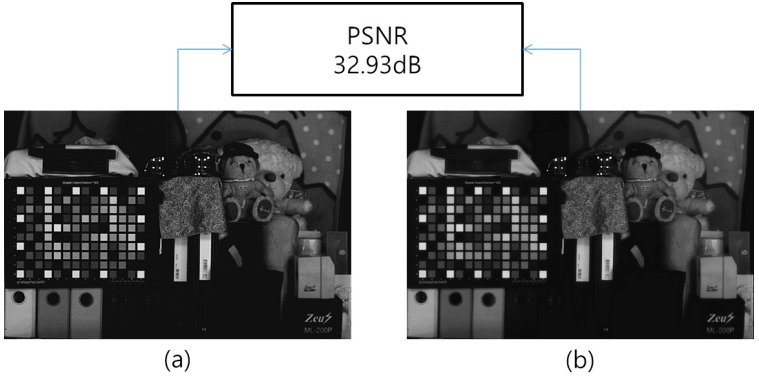
Comparison between (**a**) the optical filtered N channel image and (**b**) the estimated N channel image in visible bands.

Since the spectral response function of the RGBN filter is not defined only in the NIR band, we used a signal processing approach to estimate the NIR band response. To decompose the spectral information of the RGBN channel, the unknown value $N_{vis}$ or $N_{nir}$ must be estimated. To cope with the different characteristics of the correlation in the visible band, as well as the NIR band, we set the correlation model in each sub-band, separately. In the visible band, the RGB channel filters show different peak spectral responses, while the N channel filter covered all spectral ranges without outstanding peaks. As a result, the N channel filter response function is modeled as a linear combination of the others:

(11) $$\begin{array}{ccc} N_{vis} & = & \int_{w_{vis}}\omega_{r}\left(\lambda \right)E\left(\lambda \right)R^{\left(r\right)}\left(\lambda \right)S\left(\lambda \right)d\lambda \\ & & +\int_{w_{vis}}\omega_{g}\left(\lambda \right)E\left(\lambda \right)R^{\left(g\right)}\left(\lambda \right)S\left(\lambda \right)d\lambda \\ & & +\int_{w_{vis}}\omega_{b}\left(\lambda \right)E\left(\lambda \right)R^{\left(b\right)}\left(\lambda \right)S\left(\lambda \right)d\lambda \end{array}$$

where $\omega_{r}\left(\lambda \right)$, $\omega_{g}\left(\lambda \right)$ and $\omega_{b}\left(\lambda \right)$ represent the coefficients that show cross-correlation in the visible band. Since the spectral response of the N channel in the visible band covers a wide spectral range without an outstanding peak, those coefficients are constrained to be constant in terms of the wavelength [27]. Using the constrained weights, the intensities of the N channel in the visible band are approximated as follows:

(12) $$N_{vis}\left(i,j\right)\approx \omega_{r}\cdot R_{vis}\left(i,j\right)+\omega_{g}\cdot G_{vis}\left(i,j\right)+\omega_{b}\cdot B_{vis}\left(i,j\right)$$

where $\omega_{r},\omega_{g}$ and $\omega_{b}$ represent the visible band cross-correlation coefficients obtained by the linear transformation model:

(13) N=DC

where D is a one by three matrix describing the mapping between the RGB to N channel values. The transformation D is obtained by solving the following minimization function:

(14) $${\hat{D}}^{T}=argmin_{D^{T}}||N-D^{T}C{\left|\right|}^{2}$$

where N and C are matrices whose components are the NIR and the RGB components. Each cross-correlation coefficient could have been of any arbitrary form determined by the illuminance change and the spectral response of the sensor. As a result, the function *ω* depends not on the spectrum *λ* itself, but on the spectral response of the illuminance and the sensor. Figure 8 represents the comparison between the optical filtered N channel image in visible band and estimated N channel image in the visible band by using Equation (12).

**Figure 9:**
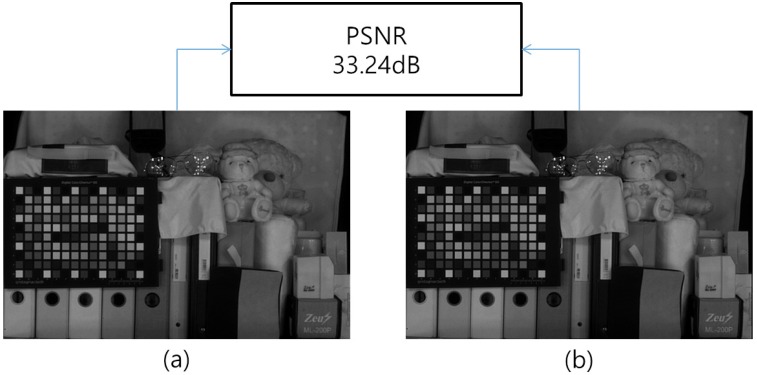
Comparison between (**a**) the optical filtered N channel image and (**b**) the estimated N channel image in NIR bands.

In the NIR band, the cross-correlation is derived more intuitively, since the RGBN filters are all pass filters where the filter responses are highly correlated in the NIR spectral range. Since there is an energy difference between the two spectral ranges in the N filter response, the cross-correlation coefficients in Equation (12) have to be modified. To cope with the different energy ratios in the visible and the NIR bands, the response of the N channel in the NIR band is:

(15) $$N_{nir}\left(i,j\right)\approx \beta_{v,n}\cdot \left(\omega_{r}\cdot R_{nir}\left(i,j\right)+\omega_{g}\cdot G_{nir}\left(i,j\right)+\omega_{b}\cdot B_{nir}\left(i,j\right)\right)$$

where $\beta_{v,n}$ is the inter-spectral correlation coefficient that considers the visible band to the NIR band energy balance. Figure 9 represents the comparison between the optical filtered N channel image in the NIR band and the estimated N channel image in the NIR band by using Equation (15).

## 4. Proposed Methods

The purpose of the proposed method is to restore the original color in the visible bands from the mixed wide band signal. However, the color restoration in the spectral domain is an underdetermined problem, as described in Equation (9). Since MSFA image sensors have additional pixels whose intensity was represented in Equation (9), we redefined this underdetermined problem with eight unknown spectral values.

From Equation (8), the observed intensity vectors of the multispectral images are represented as $C\left(i,j\right)={\left[R\left(i,j\right),G\left(i,j\right),B\left(i,j\right),N\left(i,j\right)\right]}^{T}$. To focus on the color restoration at each pixel position, we assumed that the spatially-subsampled MSFA image was already interpolated. As a result, there are four different intensities at each RGBN pixel position.

In Figure 4, the spectral response of each channel is described with the corresponding RGB and N values. The energy of the NIR band is obtained by the RGB color filters, as well as the N filter. Similarly, a large amount of the energy in the visible band is obtained by the N channel. By considering the observed multispectral intensity vector C, the spectral correlation between the channels in the visible band and the NIR band resulted in a mixture of exclusive responses in each channel, as represented in Equation (9).

From the sub-spectral band intensity mixture model, the color restoration problem is defined to find the unknown visible band intensity values $R_{vis},G_{vis},B_{vis}$ from the observed intensity values R, G, B and N, which contained the unknown NIR band intensity values and the unknown visible intensity values.

### 4.1. Color Restoration Based on Spectral Decomposition

When we spectrally decompose the N channel to the visible and NIR bands, the given N channel is represented by the RGB channel intensities in the visible and NIR bands from Equation (12) and Equation (15):

(16) $$\begin{array}{ccc} N & = & N_{vis}+N_{nir}\\ & = & \omega_{r}\cdot \left(R_{vis}+\beta_{v,n}\cdot R_{nir}\right)+\omega_{g}\cdot (G_{vis}\\ & & +\beta_{v,n}\cdot G_{nir})+\omega_{b}\cdot \left(B_{vis}+\beta_{v,n}\cdot B_{nir}\right) \end{array}$$

In Equation (16), the observed N channel is described with unknown RGB values in the visible bands and the NIR bands. Therefore, the decomposed N channel is obtained indirectly from Equation (16). Corresponding to the spectral response of the N channel, we define the artificial N channel $\hat{N}$ made by using the observed RGB channels and the visible band cross-correlation coefficients in Equation (12):

(17) $$\begin{array}{ccc} \hat{N} & = & \omega_{r}\cdot R+\omega_{g}\cdot G+\omega_{b}\cdot B\\ & = & \omega_{r}\cdot \left(R_{vis}+R_{nir}\right)+\omega_{g}\cdot \left(G_{vis}+G_{nir}\right)\\ & & +\omega_{b}\cdot \left(B_{vis}+B_{nir}\right) \end{array}$$

Since the visible band cross-correlation coefficients are designed to fit the N channel in the visible band, the estimated $\hat{N}$ value resembles the N channel filter responses in the visible band, but not in the NIR band. By using the energy difference between *N* and $\hat{N}$ in the NIR band, the observed N channel is decomposed into the two bands by subtracting the original *N* channel in Equation (16) and the artificial N channel $\hat{N}$ in Equation (17):

(18) $$\begin{array}{ccc} N-\hat{N} & = & \omega_{r}\cdot \left(\beta_{v,n}-1\right)\cdot R+\omega_{g}\cdot \left(\beta_{v,n}-1\right)\cdot G\\ & & +\;\omega_{b}\cdot \left(\beta_{v,n}-1\right)\cdot B\\ & = & \left(\beta_{v,n}-1\right)\cdot \left(\omega_{r}\cdot R_{nir}+\omega_{g}\cdot G_{nir}+\omega_{b}\cdot B_{nir}\right)\\ & = & \frac{\beta_{v,n}-1}{\beta_{v,n}}\cdot {\hat{N}}_{nir}\\ & = & K\cdot {\hat{N}}_{nir} \end{array}$$

where $K=\frac{\beta_{v,n}-1}{\beta_{v,n}}$ is a scaling factor and ${\hat{N}}_{nir}$ represents the artificial N channel in the NIR band from Equation (15). Based on Equation (18), we decompose the spectral response of the N channel into two different channels, the visible band and the NIR band. The N channel information in the NIR band is recovered from the N channel that contained the energy of the entire spectrum of the MSFA image sensor. As a result, the decomposed N channel intensities in the NIR band and the RGB channel intensities in the NIR band are estimated from the result of Equation (18).

**Figure 10:**
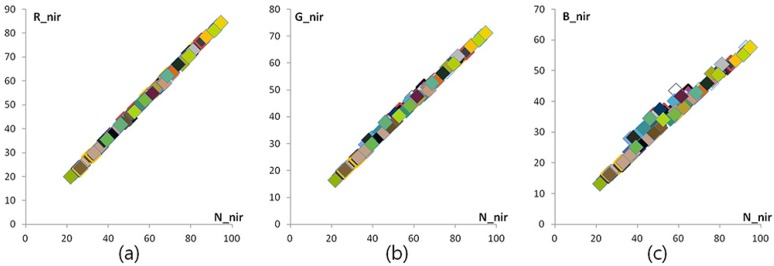
RGBN channel correlation in the NIR band: (**a**) $N_{nir}$ *vs.* $R_{nir}$; (**b**) $N_{nir}$ *vs.* $G_{nir}$; (**c**) $N_{nir}$ *vs.* $B_{nir}$.

Figure 10 shows the relationship of the RGB channel intensities and the N channel intensity of 96 color patches of the Gretag color checker SG in the NIR band. As described in Figure 10, they are asymptotically linear in the NIR band. From this linear correlation, the decomposed RGB channel in the NIR band is defined as follows:

(19) $$\begin{array}{cc} {\hat{R}}_{nir} & =\alpha_{r}\cdot {\hat{N}}_{nir}\\ {\hat{G}}_{nir} & =\alpha_{g}\cdot {\hat{N}}_{nir}\\ {\hat{B}}_{nir} & =\alpha_{b}\cdot {\hat{N}}_{nir} \end{array}$$

where $\alpha_{r}$, $\alpha_{g}$ and $\alpha_{b}$ represent the coefficients of the linear correlations between the RGB channels and the N channel in the NIR band. From the equation, the intensities of the RGB channel in the NIR band are estimated, and this color restoration model was processed with a single matrix transformation of:

(20) $${\left({\hat{R}}_{vis},{\hat{G}}_{vis},{\hat{B}}_{vis}\right)}^{T}=M\cdot {\left(R,G,B,N\right)}^{T}$$

where M is:

(21) $$M=E+\frac{1}{K}AW$$

where W is the N channel decomposition matrix, A is the RGB channel decomposition matrix and E is a 3 × 4 matrix of zeros with ones along the leading diagonal. The N channel decomposition matrix W is defined as:

(22) $$W=\left(\begin{array}{cccc} \omega_{r} & \omega_{g} & \omega_{b} & -1\\ \omega_{r} & \omega_{g} & \omega_{b} & -1\\ \omega_{r} & \omega_{g} & \omega_{b} & -1\\ \omega_{r} & \omega_{g} & \omega_{b} & -1 \end{array}\right)$$

and the RGB channel decomposition matrix is defined as:

(23) $$A=\left(\begin{array}{cccc} \alpha_{r} & 0 & 0 & 0\\ 0 & \alpha_{g} & 0 & 0\\ 0 & 0 & \alpha_{b} & 0 \end{array}\right)$$

Based on Equation (21), the unified matrix **M** is:

(24) $$\left(\begin{array}{c} {\hat{R}}_{vis}\\ {\hat{G}}_{vis}\\ {\hat{B}}_{vis} \end{array}\right)=\left(\begin{array}{cccc} \frac{\alpha_{r}\cdot \omega_{r}+K}{K} & \frac{\alpha_{r}\cdot \omega_{g}}{K} & \frac{\alpha_{r}\cdot \omega_{b}}{K} & -\frac{\alpha_{r}}{K}\\ \frac{\alpha_{g}\cdot \omega_{r}}{K} & \frac{\alpha_{g}\cdot \omega_{g}+K}{K} & \frac{\alpha_{g}\cdot \omega_{b}}{K} & -\frac{\alpha_{g}}{K}\\ \frac{\alpha_{b}\cdot \omega_{r}}{K} & \frac{\alpha_{b}\cdot \omega_{g}}{K} & \frac{\alpha_{b}\cdot \omega_{b}+K}{K} & -\frac{\alpha_{b}}{K} \end{array}\right)\left(\begin{array}{c} R\\ G\\ B\\ N \end{array}\right)$$

where $K=\frac{\beta_{v,n}-1}{\beta_{v,n}}$ is a scaling factor in Equation (18), $\omega_{r}$, $\omega_{g}$, $\omega_{b}$ are the coefficients for the linear combination in Equation (11) and $\alpha_{r}$, $\alpha_{g}$ and $\alpha_{b}$ are the coefficients that represent the linear correlation between the RGB channels and the N channel in the NIR band in Equation (19). Because Equation (24) is a combination of cascaded linear decomposition matrices W and A, the proposed color correction matrix is more flexible than the simple 3 × 4 linear color correction model. Further, because the sensor response function over the entire band is nonlinear, color correction error is inevitable when the linear color correction method is employed. Moreover, there is an energy difference between the visible and NIR bands. The spectral response of the local spectral band can be approximated to a linear model. On the basis of linear model approximation of each local spectral band, the proposed method separates the visible and NIR bands to estimate the color correction matrix and, thereby, obtain a more accurate estimation of the NIR interference in each RGB channel. Using W, the proposed method decomposes the N channel to the visible and NIR bands and uses the NIR band information obtained from W to estimate the NIR contribution in the RGB channels. The correlation between the RGB and N channels in the NIR band is estimated using A. Because the proposed method separates the visible and NIR bands to estimate the color correction matrix (CCM), it is possible to estimate the correlation between RGB and NIR in various illumination environments.

**Figure 11:**
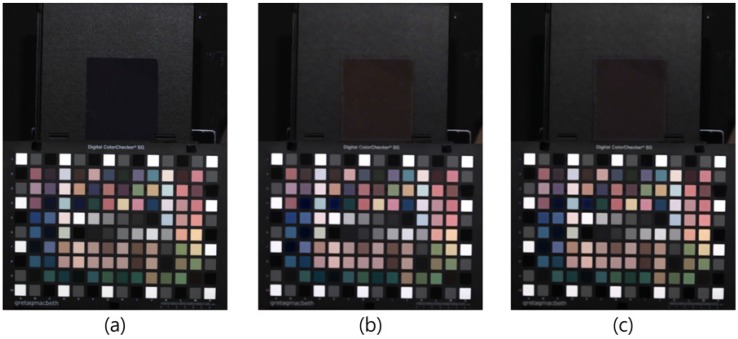
Experimental results under an incandescent lamp (300 lx). (**a**) Optical filtered visible band image; (**b**) 3 × 4 color correction method; (**c**) proposed method.

Figure 11 shows the experimental results obtained under an incandescent lamp with 300 lx illumination. Because the incandescent lamp emits an amount of spectral energy in the NIR band, we selected this lamp to show the advantage of the proposed method. By comparing Figure 11b and Figure 11c, the level of restoration of the overall colors of each color patch can be ascertained. In Figure 11a, which is the target optical filtered image, it can be seen that some color patches are slightly different. To investigate the color restoration accuracy, we calculated angular error. Table 1 shows the average angular error. From Table 1, it is clear that the proposed method restores color better than the linear 3 × 4 color correction method.

### 4.2. Low Light Conditions

**Figure 12:**
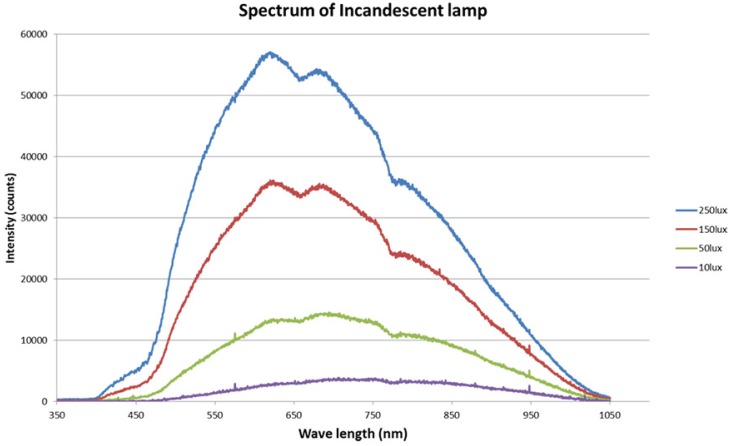
Spectrum of an incandescent lamp under various kinds of illumination (3000 K).

Because of the additional NIR band information, an IRCF-removed MSFA image sensor has advantages in low visible light conditions. From the perspective of color restoration, however, there is no advantage, since the unnecessary NIR interference to the RGB color channel does not have any visible band color information. Figure 12 represents the spectral energy distribution of an incandescent lamp with a variety of illuminance values. The correlated color temperature of the lamp is 3000 K. As illuminance decreased, the overall intensities of spectral energy decreased, too. In addition, the energy ratio between the visible band and the NIR band varied as the illuminance decreased.

Table 2 shows that decreasing illuminance increases the portion of the NIR band spectral energy under incandescent light. The numbers in Columns 2 and 3 represent the summation of the spectrum values in Figure 12. This implies that 60% of the unwanted NIR contributions in each RGB channel must be removed to obtain a natural color image under an incandescent lamp with 10 lx. Because the NIR contribution is greater than the color information in each RGB channel, it is important to estimate the NIR band spectral information precisely to prevent false color generation.

### 4.3. Two-Step Color Restoration

In general lighting situations, the proposed color restoration method based on Equation (24) can decompose the NIR contribution in each RGB channel. However, as mentioned in Section 4.2, the spectral energy distribution changed under low lighting conditions. Furthermore, the ratio between the visible band and the NIR band changed. Therefore, the estimation of the N channel in the NIR band is more important under low lighting conditions. The color restoration model in Equation (24) is based on the assumption that the spectral response of the MSFA sensor in the NIR band correlated with the spectral linearity between the RGB and N channels. However, in the 700 nm to 800 nm spectral range, there was a lack of linear correlation between the channels, except for between the R and N channels. If the spectral energy distribution of the light source shows strong energy between this nonlinear range, the spectral decomposition error of the result will increase. Because the visible band information is smaller than the NIR band under low lighting conditions, the spectral decomposition error can produce a false color result.

**Figure 13:**
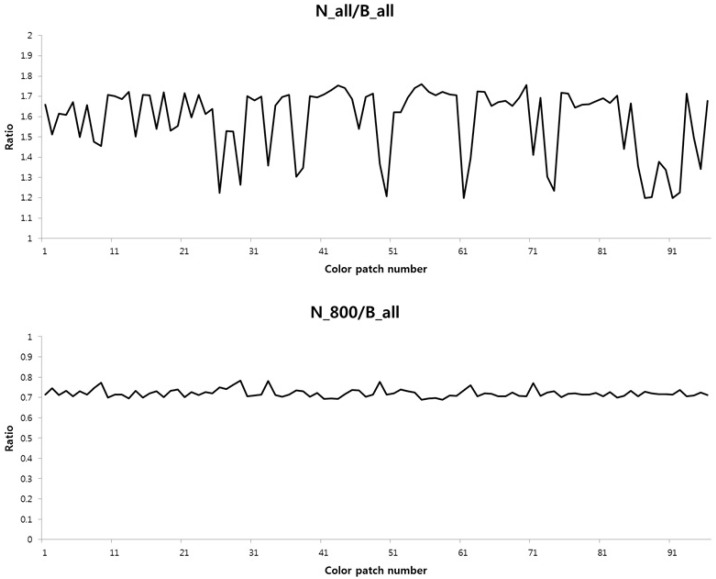
Relationship between the B channel and the N channel (incandescent lamp, 1 lx): the ratio between the B channel and the N channel in a wide spectral range (**Top**); the ratio between the B channel beyond 800 nm and the N channel in a wide spectral range (**Bottom**).

To overcome this spectral nonlinearity problem, we used a two-step color restoration method that divides the spectral range into two parts and removes the NIR band information sequentially. Figure 4 represents the two-step color restoration process. In the first step, the intensities of the RGB channel in the NIR band with a spectral wavelength range greater than 800 nm were decomposed using the B channel. In Figure 13, the ratio between the B channel and the N channel of 96 color patches of the Gretag color checker SG is represented. Since the visible band information of the B channel is quite small under low lighting conditions, there is a strong correlation between the B channel and the N channel whose wavelength is above 800 nm, as described in Figure 13.

**Figure 14:**
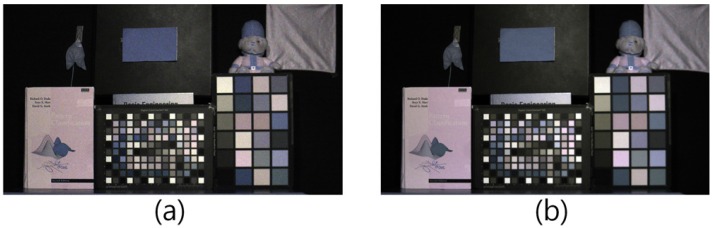
Result of the achromatic NIR band (above 800 nm) component removal (incandescent 5 lx). (**a**) Optical filtered image; (**b**) first step of the proposed method.

The N channel whose wavelength is beyond 800 nm was approximated from the B channel as follows:

(25) $${\hat{N}}_{nir}^{800}=\gamma \cdot B$$

where *γ* is the correlation coefficient between the B channel and the N channel above 800 nm. Figure 14 represents the result of Equation (25). Figure 14a is the image obtained with the optical filter, and Figure 14b is the result of the proposed method after the first step of color restoration. By comparing (a) to (b), the overall colors of the entire image were similar.

After the first step, the remaining NIR intensities in the RGB channel were removed through the spectral decomposition method as proposed in Equation (24). Based on Equation (20), the two-step color restoration model can be processed with a matrix equation as follows:

(26) $${\left({\hat{R}}_{vis},{\hat{G}}_{vis},{\hat{B}}_{vis}\right)}^{T}=M\cdot {\left(R,G,B,N\right)}^{T}-P$$

where **P** is defined as:

(27) $$P={\left(\gamma_{r},\gamma_{g},\gamma_{b}\right)}^{T}\cdot B$$

The $\gamma_{r}$, $\gamma_{g}$ and $\gamma_{b}$ values represent the correlation coefficient between the B channel and the N channel whose wavelength was above 800 nm. The proposed two-step color restoration method was applied to estimate the NIR component of the image obtained under particular illumination situations, such as low light conditions, especially the illuminance of an incandescent lamp under 5 lx. In this paper, we use the proposed method with a two-step color restoration with Equation (26) when the illuminance of the light source is under 5 lx.

From Section 2, the achromatic NIR component *δ* did not affect the hue and saturation value of the images. The achromatic NIR component is not an important part of restoring the color component. Therefore, we estimated the spectral information of the chromatic NIR band precisely after removing the achromatic NIR component *δ*.

**Figure 15:**
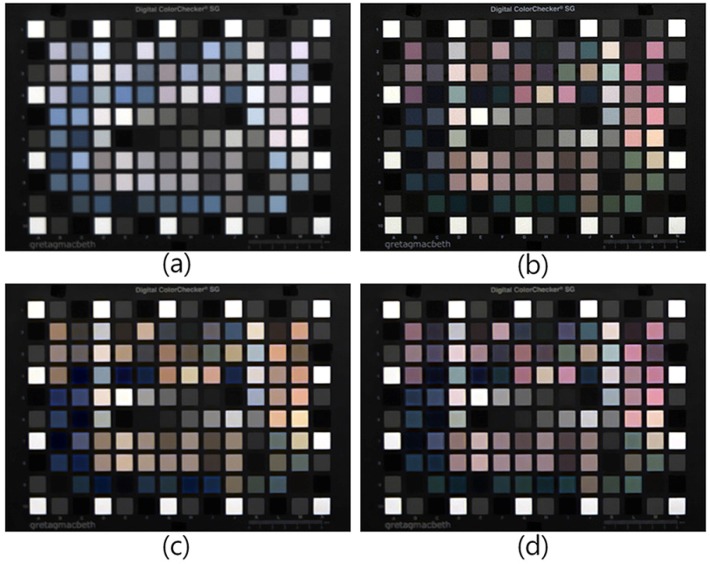
Comparison between proposed methods (incandescent, 5 lx). (**a**) Multi-spectral image; (**b**) optical filtered image; (**c**) proposed method without two-step color restoration; (**d**) proposed method with two-step color restoration.

Figure 15 represents the result of the proposed method under an incandescent lamp with 5 lx.

Figure 15a is the input image, the color of which is desaturated by additional NIR, and Figure 15b is the optical filtered visible band image. Figure 15c is the result obtained using the proposed method in Equation (24) as given in Section 4.1, and Figure 15d is the result that was obtained using the two-step color restoration described in Section 4.3. By comparing Figure 15c to Figure 15d, the overall color of Figure 15c is yellow-shifted, especially in red color patches. Since the spectral energy distribution changed under low lighting conditions, the unified color restoration model M in Equation (24) was limited in explaining the complicated nonlinear transformation. After removing the achromatic NIR band information, the only concern was the chromatic NIR band used to restore the color information. Since the unified color restoration model M handled the chromatic NIR band information, the color was successfully restored as represented in Figure 15d.

## 5. Experimental Results

**Figure 16:**
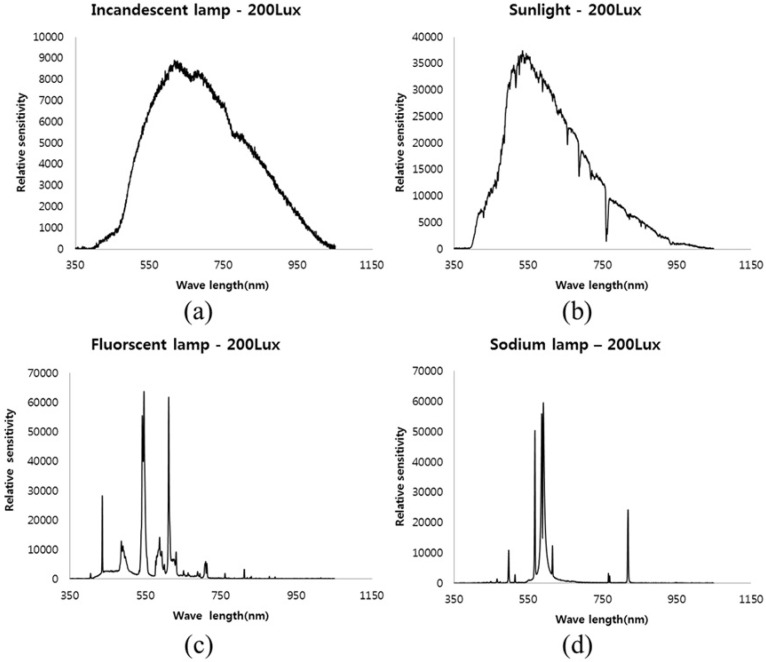
Spectral distribution of a variety of light sources. (**a**) Incandescent lamp (3000 K); (**b**) sunlight (6500 K); (**c**) fluorescent lamp (5000 K); (**d**) sodium lamp (2700 BK).

The proposed color restoration method was tested with images captured under different standard illuminations: sunlight, incandescent lamp, sodium lamp and fluorescent lamp. Since the spectrum of these light sources was spread over a wide range, we used these lights as the target illuminance values as represented by Figure 16.

As the training set for the correlation coefficients, we used 96 standard colors of the Gretag color checker SG. Because the color samples were distributed widely, these colors were used for the training set. The input multispectral image was obtained by a camera system with an RGBN image sensor without IRCF, and we used a target visible band image with an IRCF as a reference image. The 96 patches were manually segmented, and we used the average RGB of each patch. The resulting average RGB values in the input image and the reference image were used to derive a set of color restoration models in Equation (24). We also measured the XYZ of each of the 96 patches using a spectrophotometer. If an illuminance value was less than 5 lx, we used an additional optical filter that passes wavelengths beyond 800 nm to derive a set of color restoration models in Equation (27). After setting a color restoration model, the proposed method was applied to an input multispectral image without IRCF. As mentioned in Section 4.3, we used two-step color restoration when the illuminance of the light source was darker than 5 lx. In our experiment, we measured the illumination level using an illuminometer. In practical situations, the light sensor commonly used to turn on the flash light or changing to night shot mode must be installed to measure the luminance level of the illumination. The light sensor performs the simple role of determining whether the luminance level corresponds to dark or bright. When the illuminance of the light source is brighter than 5 lx, we used the color restoration model in Equation (24).

As an error criterion, the angular error was calculated. Considering the *Z* color sample entities in the training set, the angular error for the *z*-th color was defined as:

(28) $$\theta_{z}=\cos^{-1}\left(\frac{m_{z}\cdot p_{z}}{|m_{z}\left|\right|p_{z}|}\right)$$

where $\theta_{z}$ is the angular error between the target color vector $m_{z}$ and the color restoration result $p_{z}$. ‘·’ represents the inner product of two vectors, and |m| represents the magnitude of the vector m. In addition, we measured the color difference ΔE of each color sample in the CIELAB color space defined by:

(29) $$\Delta E_{ab}^{*}={\left[{\left(\Delta L^{*}\right)}^{2}+{\left(\Delta a^{*}\right)}^{2}+{\left(\Delta b^{*}\right)}^{2}\right]}^{1/2}$$

We regarded the average of ΔE as the color correction error. To convert RGB to the CIELAB color space, the RGB signals were transformed to CIE tristimulus values by using a spectrophotometer with a standard illuminant, after which the CIELAB equation was applied [28]. The tristimulus values of the illuminant were A, F and D65 with respect to the incandescent lamp, fluorescent lamp and sunlight, respectively. We used a visible band image with IRCF as a reference image that was used to compare to the input image and the result image. As comparative methods for the proposed color restoration algorithm, we implemented the least squares-based color correction method [29] and the N-to-sRGB mapping color correction method based on root-polynomial mapping [30].

**Figure 17:**
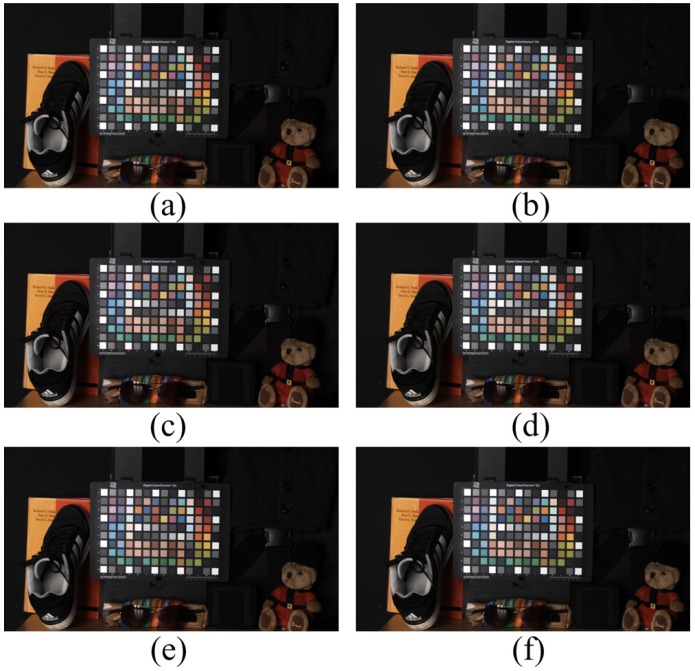
Experimental results under a fluorescent lamp (350 lx). (**a**) Input image; (**b**) optical filtered visible band image; (**c**) 3 × 3 CCM; (**d**) 3 × 4 CCM; (**e**) root-polynomial mapping; (**f**) proposed method.

Figure 17 depicts the experimental results under a fluorescent lamp with 350-lx illumination. Since the fluorescent lamp did not emit NIR, the input image in Figure 17a and the optical filtered image in Figure 17b were almost similar. Our proposed method preserved the color of the input image (Figure 17f) and the other color correction methods (Figure 17c to Figure 17e) because of the absence of NIR color distortion in the input image.

**Figure 18:**
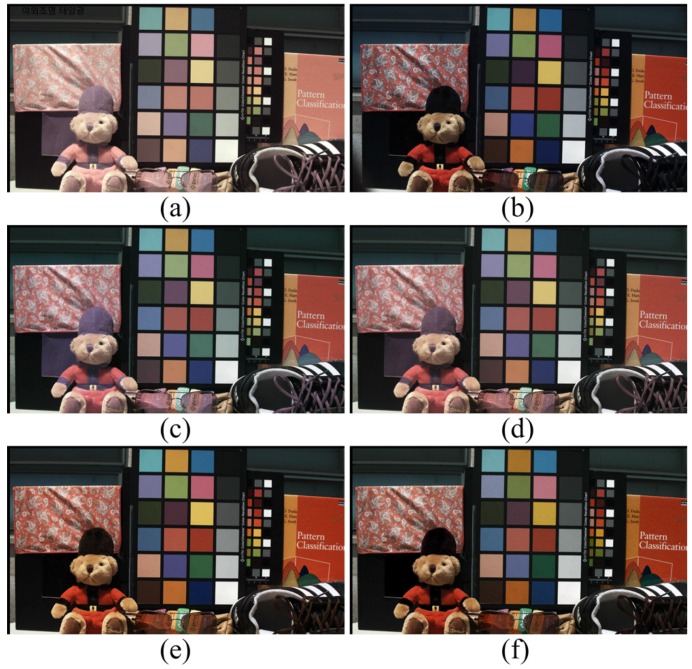
Experimental results under sunlight (400 lx) (**a**) Input image (**b**) optical filtered visible band image; (**c**) 3 × 3 CCM; (**d**) 3 × 4 CCM; (**e**) root-polynomial mapping; (**f**) proposed method.

Figure 18 shows the experimental result under sunlight, which has a wide range of spectral distribution and abundant visible band information. In this case, it was sufficient to restore color using the proposed method in Equation (24). Comparing Figure 18b and Figure 18c to Figure 18f, the resulting image of the proposed method restored the distorted color well, especially the materials with high reflectance in the NIR band. The root-polynomial mapping method in Figure 18e restored the overall colors of each color patch and black materials well. The comparison of Figure 18b,e shows that the saturation is slightly high. Since sunlight has plenty of spectral energy in visible bands, the root-polynomial mapping restores color information as well as the proposed method. To investigate color restoration accuracy, each method was compared in Table 3 and Table 4.

**Figure 19:**
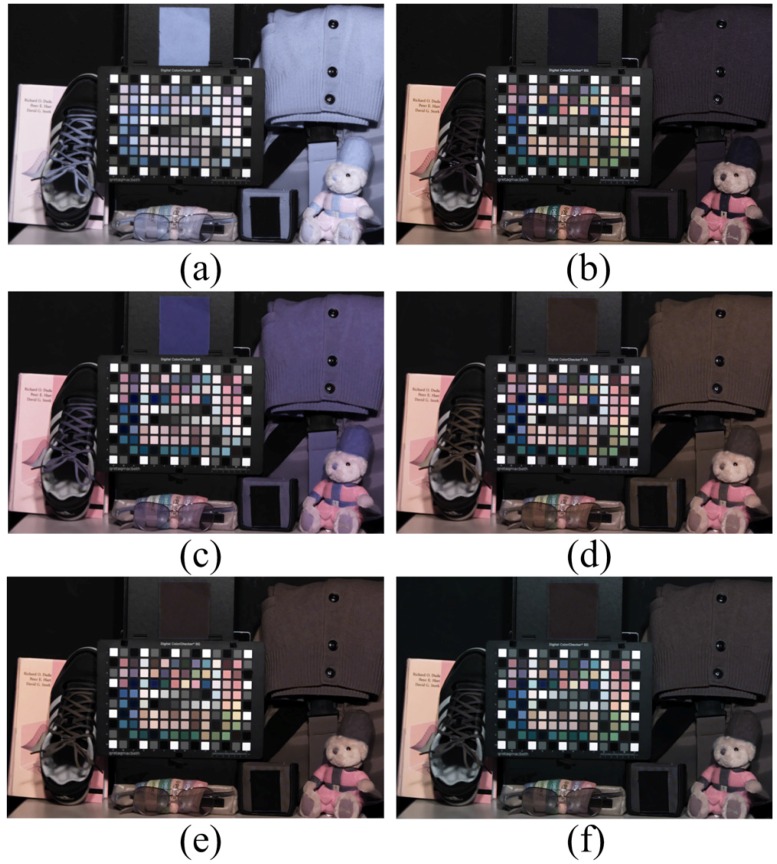
Experimental results under an incandescent lamp (200 lx). (**a**) Input image; (**b**) optical filtered visible band image; (**c**) 3 × 3 CCM; (**d**) 3 × 4 CCM; (**e**) root-polynomial mapping; (**f**) proposed method.

Another set was tested under an incandescent lamp, which emits much spectral energy in the NIR band. Figure 19a represents the multispectral image obtained under the incandescent lamp. The color channels were white balanced without considering the color degradation caused by the additional NIR; therefore, the overall colors of the image show low saturation and blue hue over much of the NIR band. Figure 19c shows the result of the conventional color correction method. When comparing Figure 19c to Figure 19b, the overall colors of each color patch and object were close to the target image. The comparison of Figure 19d to Figure 19f shows that the overall colors of each color patch and object were close to the optical-filtered visible band image (Figure 19b). However, the color of the objects with high reflectance in the NIR band, such as fabric, leaf, and so on, was slightly different. This means that the accuracy of the NIR estimation was different. Figure 19f is much closer to the visible color in Figure 19b because the proposed method separates the visible and NIR bands to estimate the color correction matrix and, thereby, obtains a more accurate estimation of the NIR interference in each RGB channel. The black colors of the fabric patch in the upper side of the image, as well as the doll’s cap and clothes were restored to their original colors successfully.

**Figure 20:**
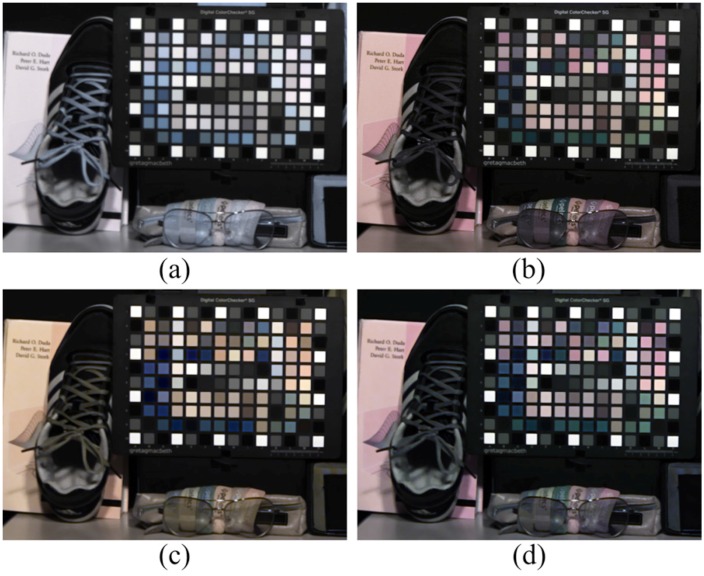
Two-step color restoration result comparison (1 lx). (**a**) Input image; (**b**) optical filtered visible band image; (**c**) proposed method without two-step color restoration; (**d**) proposed method with two-step color restoration.

As discussed in Section 4.3, the proposed two-step color restoration method is useful under particular illumination. Figure 20 represents a comparison with and without two-step color restoration under an incandescent lamp at 1 lx. Since the visible band information was less than that of the NIR band in low lighting conditions, the spectral estimation error increased. As a result, Figure 20c shows a yellow image compared to Figure 20b. With the proposed two-step color restoration method, the color of the image was successfully restored, as shown in Figure 20d. Based on this result, we tested the proposed method under low lighting situations.

**Figure 21:**
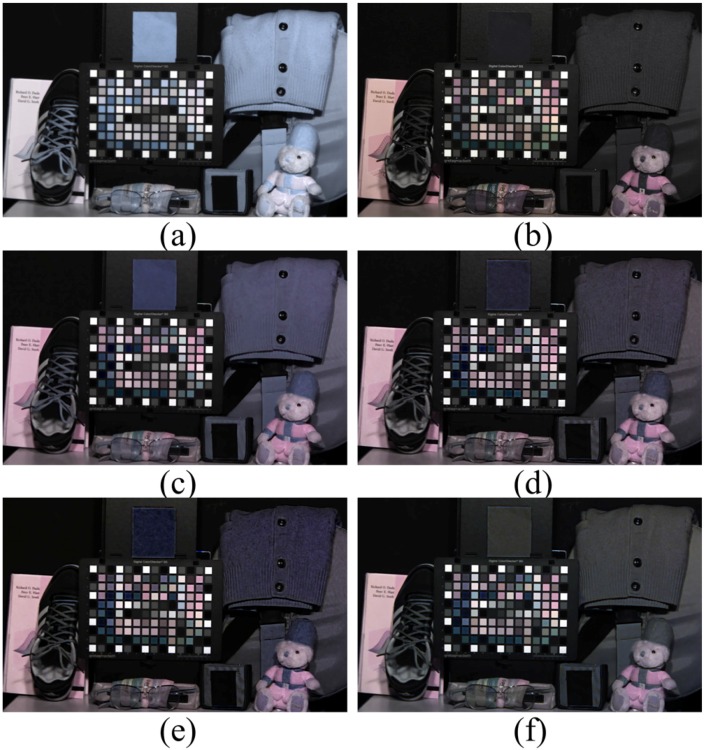
Experimental results under an incandescent lamp (1 lx) (**a**) Input image; (**b**) optical filtered visible band image; (**c**) 3 × 3 CCM; (**d**) 3 × 4 CCM; (**e**) root-polynomial mapping; (**f**) proposed method.

Figure 21 represents the experimental results under an incandescent lamp at 1 lx. This illumination emits plenty of spectral energy in the NIR band. In Figure 16, the spectrum distribution of the incandescent lamp is spread evenly over a wide range. In low lighting conditions, the lack of visible band information makes the overall saturation of the images low. Figure 21c shows that the 3 × 3 CCM-based method could not restore the overall color of the input image (Figure 21a). By comparing Figure 21d to Figure 21f, the overall colors of each color patch and object were close to the optical-filtered visible band image (b). However, the colors of black materials were not restored correctly in Figure 21d,e. Since the spectral energy of the incandescent lamp under 550 nm and the MSFA sensor response in the blue channel were low, blue information is lacking in the black area. As a result, the blue intensity was boosted during the process of color constancy. Both root-polynomial mapping and our proposed color restoration method are based on least-square linear mapping; therefore, a large amount of NIR spectral energy in low-lighting condition (see Section 4.2) must be considered. Compared to Figure 21d,e, Figure 21f shows that the proposed method restored colors satisfactorily for both the patches and for materials with high NIR component.

**Figure 22:**
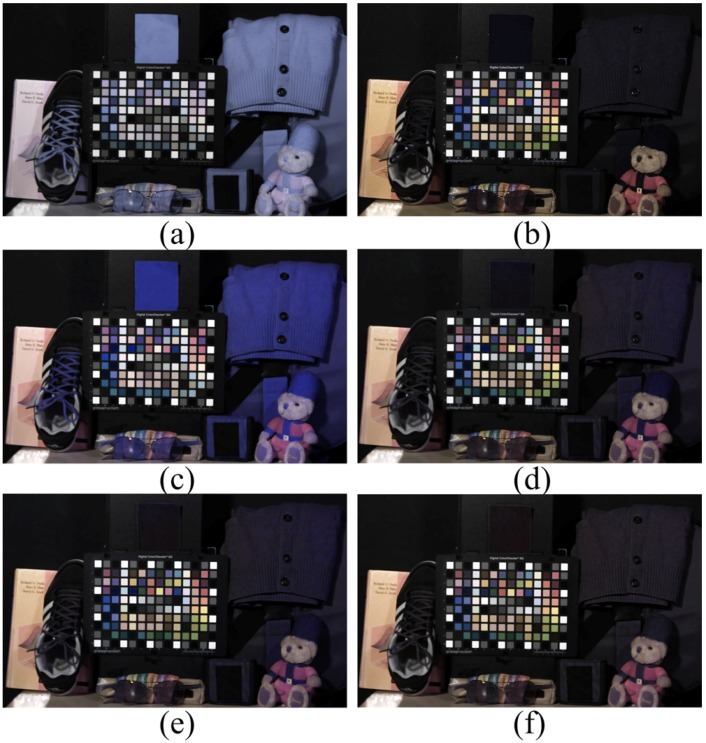
Experimental results under sodium lamp (1 lx) (**a**) Input image; (**b**) optical filtered visible band image; (**c**) 3 × 3 CCM; (**d**) 3 × 4 CCM; (**e**) root-polynomial mapping; (**f**) proposed method.

Figure 22 represents the experimental results under a sodium lamp at 1 lx. Figure 22c shows that the 3×3 CCM-based method could not restore the overall color of the input image (Figure 22a). The spectrum distribution of the sodium lamp is concentrated at a particular wavelength at 830 nm, as shown in Figure 16. In this case, the sensor spectral response of the local spectral band can be approximated to a linear model. For this reason, the experimental results in Figure 22d to Figure 22f show high restoration performance visually. To investigate the color restoration accuracy, each method was compared in Table 3 and Table 4.

Table 3 and Table 4 show the average angular error and the color difference with a variety of light sources. The performance of the proposed method was confirmed visually for materials with high reflectance in the NIR band. However, the performance of the proposed method for various colors in the color chart and substances had to be measured. Table 3 shows the amount of angular error, where our proposed method outperformed other methods. Since the color of the input image was severely distorted, the angular error between the input image and the optical filtered image was significantly high. After the application of color correction methods, the average angular errors were reduced, and the performance of the proposed method was better than that of the conventional methods. Similarly, the color difference in Table 4 shows that the color correction results obtained with the proposed method were better compared to the another methods.

**Figure 23:**
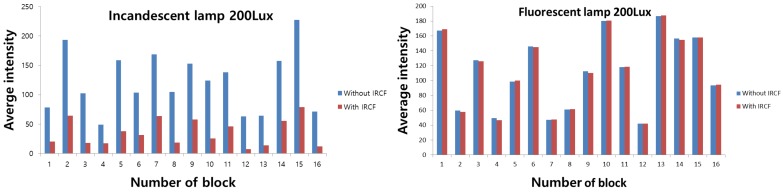
Sensitivity boosting provided by the NIR information.

In addition, to calculate the gain advantage provided with NIR information, we measured the intensities of the image obtained in various illuminations with or without IRCF. Figure 23 represents the sensitivity boosting provided by the NIR information. To measure the additional intensities, the image is divided into 16 sections. After that, the intensities are averaged in each section. As shown in Table 5 and Figure 23, the sensitivity was boosted by 10 dB without IRCF under an incandescent lamp. On the contrary, because the fluorescent lamp does not emit an NIR component, there is no gain advantage.

## 6. Conclusions

In this paper, a color restoration algorithm for an IRCF-removed MSFA image sensor in low light conditions was proposed. In the proposed method, the color degradation caused by the spectral composition of the visible and NIR band information was mainly considered. For the spectrally-degraded color information with RGB channels, the spectral estimation and spectral decomposition method were proposed to remove additional NIR band spectral information. Based on the channel estimation when considering the nonlinearity of the spectral response function of the MSFA sensor in low light conditions, the channel approximation using the B channel is for two-step color restoration. Based on the filter correlation, the inter-channel correlation on the visible and NIR band were assumed, respectively. When the N channel was decomposed into visible and NIR band information, the RGB channel in the visible band was finally restored with spectral decomposition. The experimental results show that the proposed method effectively restored the visible color from the color-degraded images caused by IRCF removal.

## Figures and Tables

**Table 1:** Average angular error (×10${}^{-2}$). CCM, color correction matrix.

	3 × 4 CCM	Proposed Method
Incandescent (300 lx)	5.12	4.17

**Table 2:** Relationships between illuminance and the portion of the NIR band spectral energy.

Illuminance	Visible Band	NIR Band	Portion of the NIR Band (%)
250 lx	57,978.6	45,337.7	**43.8**
150 lx	26,833.2	23,587.7	**46.7**
50 lx	8045.7	8847.2	**52.3**
10 lx	1347.9	2042.7	**60.2**

**Table 3:** Average angular error.

	Average Angular Error (×10${}^{-2}$)				
	Input Image	3 × 3 CCM	3 × 4 CCM	Root-Polynomial	Proposed
fluorescent (350 lx)	0.77	0.80	0.77	0.78	0.77
sunlight (400 lx)	6.97	2.93	2.27	1.98	1.53
incandescent (200 lx)	28.73	7.79	5.31	5.05	4.53
incandescent (1 lx)	29.94	8.71	5.88	6.59	4.89
sodium (1 lx)	28.94	5.99	3.15	3.13	3.13

**Table 4:** Average color difference, ΔE.

	Average Color Difference ΔE				
	Input Image	3 × 3 CCM	3 × 4 CCM	Root-Polynomial	Proposed
fluorescent (350 lx)	0.98	1.12	1.06	1.04	1.04
sunlight (400 lx)	15.66	10.97	9.83	8.16	7.50
incandescent (200 lx)	20.32	8.62	4.98	4.55	4.19
incandescent (1 lx)	22.28	8.18	6.45	7.24	5.07

**Table 5:** Average intensity value with or without IRCF in various illuminations.

Illumination	With IRCF	Without IRCF	Sensitivity Gain (dB)
Incandescent	35.6	122.3	10.71 dB
Fluorescent	112.7	112.5	0.01 dB
